# Urban green space use during a time of stress: A case study during the COVID‐19 pandemic in Brisbane, Australia

**DOI:** 10.1002/pan3.10218

**Published:** 2021-05-26

**Authors:** Violeta Berdejo‐Espinola, Andrés F. Suárez‐Castro, Tatsuya Amano, Kelly S. Fielding, Rachel Rui Ying Oh, Richard A. Fuller

**Affiliations:** ^1^ School of Biological Sciences The University of Queensland Brisbane Qld Australia; ^2^ Instituto de Investigación de Recursos Biológicos Alexander von Humboldt Bogota Colombia; ^3^ School of Communication and Arts The University of Queensland Brisbane Qld Australia

**Keywords:** COVID‐19, ecosystem services, nature‐based, pandemic, resilience, stress, urban green spaces

## Abstract

1. Spending time in nature is one potential way to cope with the negative physical and psychological health impacts from major stressful life events. In 2020, a large fraction of the global population was impacted by restrictions to contain the spread of the COVID‐19 outbreak, a period characterised by marked health risks and behavioural changes. Here we explore whether people responded to this stressor by spending more time in nature and investigate the reasons for any changes.
2. We surveyed 1,002 people in Brisbane, Australia in 2020, to measure the change in use of green space during the restrictions period and benefits people associated with visiting them.
3. About 36% of participants increased their urban green space use, but 26% reduced it, indicating a great deal of flux. Furthermore, 45% of the previous non‐users of urban green space began using it for the first time during the restrictions period. Older people were less likely to increase their green space use and those with a backyard were more likely to increase their use of green spaces.
4. Participants' change in use occurred regardless of the amount of green space available in close proximity to their households. In addition, we did not find a relationship between nature‐relatedness and change in use.
5. People's reasons for green space use shifted during the pandemic‐related restrictions period, with many emphasising improvement of personal well‐being rather than consolidating community capital. Most participants indicated an increase in the importance of the psychological and physical benefits obtained from urban green spaces.
6. We conclude that increased urban green space use during moments of stress such as the COVID‐19 pandemic has the potential to ameliorate some of the negative effects of the stressor, but that the capacity and desire to spend more time in green space varies markedly across society. Sufficient urban green space provision for all sections of society will maximise the opportunity to employ a nature‐based coping mechanism during times of personal or community stress.

## Abstract 2

A free Plain Language Summary can be found within the Supporting Information of this article.

## Resumen

1. Pasar tiempo en la naturaleza es una forma de contrarrestar el impacto físico y psicológico que tiene los eventos estresantes en nuestras vidas. En el 2020, una gran porción de la población mundial fue impactada por las restricciones impuestas por la propagación del COVID‐19, un periodo que se caracterizó por notables riesgos para la salud y cambios en el comportamiento de las personas. Aquí exploramos si las personas respondieron a este evento estresante pasando más tiempo en la naturaleza y también investigamos las razones asociadas a cualquier cambio.
2. Encuestamos a 1002 personas en Brisbane, Australia, en el 2020, para medir el cambio en el uso de los espacios verdes públicos durante la cuarentena ocasionada por la pandemia y los beneficios que la gente ha asociado a su visita.
3. Alrededor del 36% de los encuestados aumentaron el uso de los espacios verdes públicos urbanos y el 26% lo redujo, lo que indica un cambio en la concepción del uso durante la pandemia. Esto se confirma ya que un 45% de los encuestados que no han usado espacios verdes públicos previo a la cuarentena, lo utilizaron por primera vez. Los adultos mayores fueron menos proclive a aumentar el uso de espacios verdes y los encuestados que poseían un patio en sus hogares, tendieron a un mayor uso de los espacios verdes.
4. Independientemente a la cantidad disponible de espacios verdes públicos en la proximidad de los hogares de los participantes, se ha producido un cambio en las visitas a espacios verdes públicos. Además, no encontramos una relación entre la conexión con la naturaleza de los participantes y el cambio de uso de espacios verdes públicos.
5. Durante la cuarentena, los participantes han cambiado las razones por las cuales utilizaron espacios verdes públicos urbanos. Muchos participantes hicieron hincapié en los beneficios al bienestar personal en lugar de aquellos beneficios comunitarios. Por ejemplo, la mayoría de los participantes indicaron un aumento de la importancia de los beneficios psicológicos y físicos obtenidos por el uso de espacios verdes públicos urbanos.
6. Concluimos que, en momentos de estrés, como por ejemplo la pandemia COVID‐19, un aumento en el uso de espacios verdes tiene el potencial de mejorar algunos de los efectos negativos del estrés, pero que el deseo y la habilidad de pasar más tiempo en los espacios verdes varía notablemente en la sociedad. Proveer suficientes espacios verdes públicos para todos los sectores de la sociedad, maximizaría la oportunidad de emplear un mecanismo para afrontar los estreses personales o comunitarios, basado en la naturaleza.

## 要旨

1. 自然の中で時間を過ごすことは、人生における主要なストレスの多い出来事から受ける身体的・心理的な健康への悪影響に対処する一つの方法だと考えられる。世界人口の大半がCOVID‐19の発生拡大を抑制するための生活上の制限によって影響を受けた2020年は、顕著な健康リスクが発生し、人々の行動も大きく変化した特徴的な期間であった。本研究では、人々が自然の中でより多くの時間を過ごすことでこのストレス要因に対応したかどうかを調べ、また変化の理由を調査した。
2. 2020年にオーストラリアのブリスベンで1002人を対象に調査を行い、制限期間中の緑地利用の変化と、緑地への訪問がもたらす恩恵を測定した。
3. 結果には大きなばらつきがあり、約36%の調査回答者が制限期間中に都市部の緑地利用を増やしたが、26%は逆に利用を減らしていた。また、これまで都市緑地を利用していなかった回答者の45%が、制限期間中に初めて都市緑地を利用したことが明らかになった。また、高齢者は緑地利用を増やす傾向が少なく、自宅に裏庭がある回答者の方が緑地利用を増やす傾向があった。
4. 一方で、回答者の自宅近隣に存在する緑地量や自然とのつながりの強さは、緑地利用の変化程度と関係が見られなかった。
5. パンデミックに関連した制限前に比較して人々が緑地を利用する理由は変化し、多くの回答者は地域社会における便益よりも個人の福利向上を緑地利用の理由として重視していた。ほとんどの回答者は、制限期間中に都市の緑地から得られる心理的・身体的な恩恵の重要性が増したと回答した。
6. 結論として、COVID‐19パンデミックのようなストレス時に都市の緑地利用を増やすことで、ストレス要因による負の影響の一部を改善できる可能性があるものの、実際にどれだけ緑地利用を増加させられるか、また増加させたいかは人によって著しく異なることが明らかになった。社会の全ての人に十分な都市緑地を提供することで、個人や地域社会レベルのストレスに対して自然に基づく対処メカニズムを採用する機会を最大化することができるだろう。

## 1 INTRODUCTION

In the first quarter of 2020, the global community undertook a series of actions designed to decelerate the spread of the novel coronavirus SARS‐CoV‐2 (causing coronavirus disease 2019, hereafter COVID‐19). Many of these actions comprised lockdown strategies aimed at drastically reducing people's mobility (Google, 2020). The fast‐moving situation combined with poor or inadequate provision of information caused a number of human behavioural changes (Goldman, 2020), along with wide‐ranging psychological impacts (Brooks et al., 2020). For instance, in one survey where people reported spending 20–24 hr per day in quarantine, more than half reported the psychological impacts of COVID‐19 as moderate to severe and one‐third reported moderate‐to‐severe anxiety (Wang et al., 2020). Urban green spaces provide a broad range of benefits to human health and well‐being that could potentially ameliorate some of these acute stresses. The pandemic has provided a unique opportunity not only to examine changes in the urban green space use under this specific set of conditions but also to gather information about people's reasons for visiting green spaces.

Health and well‐being benefits derived from urban nature can reduce many of the stresses associated with urban living (Houlden et al., 2018; Keniger et al., 2013; Twohig‐Bennet & Jones, 2018; Zhang et al., 2017). More broadly, nature experiences can play a pivotal role for mental health during stressful life events. For instance, Marselle et al. (2019) found that stressful life events were associated with an increase in perceived stress, depression, and a decrease in positive affect and mental well‐being, while nature group walks were associated with a decrease in perceived stress and depression and an increase in positive affect and mental well‐being. Furthermore, experiencing nature was key in rehabilitating individuals who had been severely affected by a life crisis, such as a divorce, bereavement or other severe loss (Ottosson & Grahn, 2008). Also, van den Berg et al. (2010) found that among patients who suffered a range of stressful life events, those who had access to large amounts of green space within 3 km of their residence reported better perceived mental and general health, compared to patients with low amount of green space in this radius. Several studies during the COVID‐19 pandemic‐related lockdown found significant outcomes associating physical and mental health with urban nature. For instance, Frühauf et al. (2020) concluded that moderate physical activity outdoors during periods of mobility restrictions, such as exercise in nearby urban forests, can deliver important benefits to physical and mental health. Other research shows that the relative abundance of indoor and outdoor nearby nature was associated with lower depression/anxiety symptoms and lower depression/anxiety rates in a cohort of students in Bulgaria (Dzhambov et al., 2020). Furthermore, Soga et al. (2020) found that the use of green space and the existence of green window views from within the home were associated with increased levels of self‐esteem, life satisfaction and subjective happiness and decreased levels of depression, anxiety and loneliness. Although there are many studies of individual effects of green space use on various aspects of health and well‐being, the global pandemic of COVID‐19 presents an opportunity to measure the role of green spaces as a nature‐based coping mechanism during a stressful life event that was simultaneously experienced by a large portion of the global community.

Urban green spaces could contribute to enhancing individual and community resilience during stressful times. Psychological resilience is the process of an individual to adapt to adversity, trauma, tragedy, threats or significant sources of stress (American Psychological Association, 2012). Examples of stressful events include the death of a family member or close friend, illness or injury, unemployment, financial crises, separation or break‐up of relationships, interpersonal or legal problems, and losses (Brugha et al., 1985). On the other hand, social resilience is a community's ability to recover from and/or respond positively to external stresses and disturbances as a result of social, political or environmental change (Adger, 2000). Examples of external stresses to a community range from natural catastrophes and financial crises to global pandemics (Fernández‐Prados et al., 2021; Khalili et al., 2015; Serban & Talânga, 2015). Nature experiences have been suggested to promote both individual‐ and community‐level resilience (Buikstra et al., 2010; Tidball, 2012; Zautra et al., 2010). The COVID‐19 pandemic has had significant impacts on public physical and psychological health (Huang & Zhao, 2020; Salari et al., 2020) and studies have shown that a regular dose of nature can contribute to the improvement of a wide range of markers of well‐being during the pandemic (Frühauf et al., 2020; Soga et al., 2020). This information is key for understanding the extent to which urban green spaces contribute to individual and community resilience.

Here, we investigate whether and how urban green space use changed during the COVID‐19 pandemic. We focused on a broad cross‐section of urban residents in the city of Brisbane, Australia during the period of the COVID‐19‐related mobility restrictions. We (a) document changes in the pattern and frequency of visitation to urban green spaces, (b) determine whether any change is associated with a change in people's perceptions of the benefits provided by green spaces, (c) explore the factors that predicted change in use of urban green spaces and (d) evaluate whether people with a stronger orientation towards nature were more likely to increase their use of urban green spaces. Our results highlight the importance of urban green spaces for urban residents during this time of major stress, providing a range of health, recreational and other benefits. We thus emphasise the need to ensure sufficient urban green space provision for all sections of society, as this will maximise the opportunity to employ a nature‐based coping mechanism during times of personal or community stress.

## 2 METHODS

### 2.1 Study area

We conducted our study in Brisbane, the state capital of Queensland, Australia. The city has an estimated human population of 1.25 million residents, approximately 4.7% of Australia's population with a population density of 842 individuals per km^2^ (Brisbane City Council, 2020a). Restrictions on human movement and interaction to slow the spread of COVID‐19 included the closure of schools and universities, indoor fitness and sports facilities, and all food, drink and cultural venues; the practice of social distancing and good hygiene; work from home where possible and to leave home only for essential trips. These restrictions were introduced on 23 March 2020 and significantly relaxed on 2 May 2020 (henceforth termed ‘restrictions period’; Australian Government Department of Health, 2020a). One of the few reasons where people were permitted to leave home was recreation in a public space such as an urban green space, with such trips being restricted to no more than two people per household and limited to their immediate neighbourhood (Australian Government Department of Health, 2020a). Brisbane's green spaces network comprises more than 2,100 urban parks and picnic grounds parks, pocket parks, riversides, botanic gardens, nature reserves and beaches. These green spaces are widespread across the city containing remnant and non‐remnant vegetation cover that provides habitat and connectivity to 83 different vegetation communities and over 2,300 species of wildlife and native plants (Brisbane City Council, 2020b, 2020c; Shanahan et al., 2014).

### 2.2 Survey and data collection

We quantified the change in participants' use and perception of urban green spaces before and during the restrictions period, and we also measured nature‐relatedness at the time of the survey. To reduce bias from those who opt‐in to participate, we chose to use *probabilistic sampling* (individuals in the population have a known non‐zero chance of being selected through the use of a random selection procedure) as opposed to *convenience sampling* methods (the probability that every individual included in the sample cannot be determined, or it is left up to each individual to choose to participate; Fricker, 2008). The survey was delivered online to participants by a market research company in June 2020, in accordance with the Institutional Human Research Ethics Approval, approval number 2020001073 (the full survey is provided in the Supporting Information). All participants were at least 18 years old and provided written consent by checking a box indicating their agreement to participate in the survey.

Participants were invited to complete the survey according to four nested stratification criteria that ensured the sample reflected a range of Brisbane's demographic groups, broad socio‐economic spread and an even spatial distribution across the city. The stratification rules were as follows: (a) an equal number of males and females, (b) an equal number of participants above and below 45 years of age, (c) the income quartiles reflected those from the total Brisbane population and (d) participants' location of residence was evenly distributed across Brisbane (North/South/East/West side of Brisbane). Participants also provided information on the number of people living at home (specifying any school‐aged children; Schipperijn et al., 2010, and the primary language spoken at home as a proxy for ethnicity; Kimpton, 2017). Furthermore, participants provided either their exact address, the address to the nearest 10 houses or the location of the street corner closest to their home. This information was used to obtain an objective measure of green space availability and proximity within a 300 m radius around each participant's residence. We chose 300 m as this distance has been widely recommended as a target for public access to local urban green spaces (Annerstedt van den Bosch et al., 2015). To achieve this, we geolocated residences using either (a) the exact address provided by participants or in cases where only the name of one street of the residence was given and (b) the midpoint of the street provided. Then, we used a map of public green spaces provided by Brisbane City Council (Brisbane City Council, 2020d) to calculate public green space coverage within the 300 m buffer. For those participants who did not provide their address, we calculated green space availability by averaging the amount of green space within the radius of all the residences sharing the same postcode.

### 2.3 Use and perception of urban green spaces

To ensure that all participants had a shared understanding of urban green spaces, a definition was provided at the start of the survey, which described green spaces as including urban parks, bushlands, picnic areas, riversides and beaches across Brisbane. The sampling design was randomised to avoid biases towards a particular type of green space with certain characteristics. Participants were asked to report whether they had visited an urban green space before and during the restrictions period, specifying their frequency of use in both time points (*never, once every two weeks, once a week, 2–3 days a week, 4–5 days a week and 6–7* *days* *a week*). We also asked participants to rate their reasons for using urban green spaces (*physical health benefits, reduction of stress, reduction of anxiety, reduction in depression, connection to nature, connection to spiritual side, appreciation of the environment, family togetherness, provision of clean air and sense of community*) and whether each of these had increased or decreased in importance during the restrictions period using a 5‐point Likert scale (*1 = much more important, 5 = much less important*). For the analysis of the change on perceptions of benefits provided by urban green spaces, we constructed a new variable named ‘*psychological well‐being benefits*’ by summing up three of the perceptions provided in the survey questionnaire: reduction of stress, reduction of anxiety and reduction in depression. These three states are known to be strongly related to psychological distress under ‘normal’ circumstances and also during the COVID‐19 pandemic (Lee et al., 2020; Rahimnia et al., 2013). To understand whether people with access to a backyard showed a different change in their green space use, participants were also asked to report whether they had access to a backyard in their place of residence.

### 2.4 Change in frequency of use of urban green spaces

We calculated the change in frequency of green space use by subtracting the category of frequency of use (*never* (1), *once every two weeks* (2), *once a week* (3), *2–3* *days* *a week* (4), *4–5* *days* *a week* (5) and *6–7* *days* *a week* (6)) during the restrictions period from the usage before the restrictions period. Positive numbers (1–5) represent an increase in the frequency of use, whereas negative numbers (−1 to −5) represent a decrease in the frequency of use. Zero denotes no change in the frequency of use.

### 2.5 Nature‐relatedness

We hypothesised that people more oriented to nature would be more likely to use urban green spaces than those with a weaker connection to nature (Lin et al., 2014). Thus, we measured participants' levels of connectedness with the natural world using the Nature‐Relatedness Scale (Nisbet et al., 2009). This validated scale consists of 21 statements representing three factors: (a) NR‐Self, an internalised identification with nature; (b) NR‐Perspective, an external environmental worldview and (c) NR‐Experience, a measure of the level of comfort with, and desire to be out in, nature. These can be summarised, respectively, as assessing the affective, cognitive and experiential aspects of a person's connection to nature. Participants used a 5‐point Likert scale to rate their level of agreement (1 = *strongly disagree*, 5 = *strongly agree*) with each of the 21 statements, with several of the statements being reverse coded. Responses to all 21 statements can be averaged to create an overall NR‐Average measure of connection to nature (Nisbet et al., 2009) with a higher score indicating a stronger connection to nature.

### 2.6 Statistical analysis

To examine the reported change in frequency of use from before to during the restrictions period, we first conducted a cumulative link mixed‐effects model (ordinal regression) with the frequency of green space use as the response variable, a binary indicator of use before and during the restrictions period as the explanatory variable, and participant ID as a random factor.

To investigate the association between six explanatory variables (gender, age, income, nature‐relatedness score, backyard access and green space availability within a radius of 300 m) and the change in frequency of green space use, we used generalised linear mixed models. We used generalised linear mixed models with a Gaussian distribution for this analysis because the frequency distribution of the response variable (change in the frequency of use of urban green spaces) approximated a normal distribution. As a result of the unprecedented circumstances of the COVID‐19 pandemic, insufficient knowledge existed of people's behaviour under study to generate a clear hypothesis of what would drive the change of urban green space use. Therefore, we used an explorative approach that generates rather than tests hypothesis about unknown relationships (Symonds & Moussalli, 2011). For this, we generated a global model with all the explanatory variables, and then compared models for all possible explanatory variable subsets using Akaike information criterion (AIC; Burnham & Anderson, 2002). We also tested global models with interactions between explanatory variables. We used participants' postcode as a random factor, aggregated into groups of five adjacent postcodes to increase variance among data points. All continuous explanatory variables were standardised. Also, all continuous explanatory variables were log‐transformed (where appropriate) to meet assumptions of normality. Prior to running the global model, we tested for correlations between explanatory variables using Spearman's rank correlations. Since green space availability and the nearest green space proximity were highly correlated (*r* = 0.67), we only used green space availability for further analyses. We then selected the models that accounted for 85% of the cumulative Akaike weights as the best‐ranked models and compared them using their delta‐AIC values. To investigate the sensitivity of the results to the choice of this model type, we also examined the association between all six explanatory variables and change in frequency of use using the cumulative link mixed‐effects model (ordinal regression; Table S1).

All statistical and spatial analyses were carried out in R v1.2.5033 (RStudio Team, 2020). We used the following packages in R: *ordinal* (Christensen, 2019) for cumulative link mixed‐effects modelling, glmmtmb (Brooks et al., 2017) for generalised linear mixed modelling, car (Fox et al., 2020) for regression companion analyses, mumin (Barton, 2020) for multi‐model inference, hmisc (Harrell & others, 2021) to generate correlation matrices, and landscapemetrics (Hesselbarth et al., 2019), fasterize (Ross, 2020), rgdal (Bivand et al., 2020), and raster (Hijmans, 2020) for spatial analyses.

## 3 RESULTS

A total of 1,002 people responded to the survey, comprising 503 identifying as female, 497 as male, and 2 as other gender. Table 1 illustrates how participants were stratified by age, income and other socio‐demographic characteristics.

**TABLE 1:** Socio‐demographic characteristics of the participants and change of use of green space use calculated by subtracting the category of frequency of use (*never, once every two weeks, once a week, 2–3 days a week, 4–5 days a week and 6–7* *days* *a week*) during the restrictions period from the usage before the restrictions period. A positive number represents increased use, a negative number represents decreased use and zero denotes no change in frequency of use

	*n*	%	Number (%) increasing use	Number (%) decreasing use	Number (%) no change in use
Gender					
Male	497	49.6	170 (34.2)	120 (24.2)	207 (41.6)
Female	503	50.2	195 (38.8)	143 (28.4)	165 (32.8)
Other	2	0.2	0	0	2 (100)
Age					
18–25	124	12.4	61 (49.2)	30 (24.2)	33 (26.6)
26–35	234	23.4	101 (43.2)	54 (23.1)	79 (33.7)
36–45	173	17.3	80 (46.2)	41 (23.7)	52 (30.1)
46–55	129	12.9	39 (30.2)	49 (38)	41 (31.8)
56–65	138	13.8	30 (21.7)	35 (25.4)	73 (52.9)
66–70+	201	20.1	52 (25.9)	54 (26.9)	95 (47.2)
Income (per annum)					
$104,000 or more	0	0	0	0	0
$78,000–$103,999	122	12.8	39 (32)	36 (29.5)	47 (38.5)
$65,000–$77,999	140	14.7	57 (40.7)	27 (19.3)	56 (40)
$41,600–$64,999	202	21.3	83 (41.1)	46 (22.8)	73 (36.1)
$20,800–$41,599	201	21.2	72 (35.8)	56 (27.9)	73 (36.3)
$1–$20,799	255	26.8	82 (32.1)	77 (30.2)	96 (37.6)
No income	30	3.2	15 (50)	5 (16.7)	10 (33.3)
Languages spoken at home					
Only English	857	85.5	299 (34.9)	231 (26.9)	327 (38.2)
Others	145	14.5	66 (45.5)	32 (22.1)	47 (32.4)
Household with backyard					
Yes	836	83.4	309 (37)	211 (25.2)	316 (37.8)
No	166	16.6	56 (33.8)	52 (31.2)	58 (35)
Household with school‐aged children					
Yes	218	21.8	92 (42.2)	57 (26.1)	69 (31.7)
No	784	78.2	273 (34.8)	206 (26.3)	305 (38.9)
Nature‐relatedness score					
1 > 2	4	0.39	1 (25)	2 (50)	1 (25)
2 > 3	181	18.06	76 (42)	49 (27.1)	56 (30.9)
3 > 4	588	58.68	205 (34.9)	167 (28.4)	216 (36.7)
4 > 5	229	22.85	83 (36.2)	45 (19.7)	101 (44.1)
5	0	0	0	0	0
Total	1,002	100	365 (36.5%)	263 (26.2%)	374 (37.3%)

### 3.1 Change in use of urban green spaces

There was a marked change in the pattern of urban green space use during the restrictions period, with 62.7% of survey participants changing their use of urban green spaces relative to the time before the restrictions were introduced. About 36.5% (*n* = 365) of people increased their use, 26.2% (*n* = 263) decreased and 37.3% (*n* = 374) did not change their frequency of green space use relative to the period before the restrictions were imposed (Table 1). Across all participants, 12.8% (*n* = 129) used urban green spaces daily before the restrictions period, but this increased to 19.2% (*n* = 193) during the restrictions period. There was also an increase in the number of people who did not use green spaces at all from 8.5% (*n* = 85) before the restrictions period to 12.3% (*n* = 124) during the restrictions period.

The increase in participants' use of urban green spaces during the restrictions period compared to before the restriction period was statistically significant (estimated coefficient = 0.441; standard error = 0.086, *p* < 0.001). The proportion of people who increased green space use during the restrictions period was greater than those who decreased across almost all demographic groups (Table 1), with the important exception of people over the age of 45, who mostly either maintained or reduced their level of green space use.

### 3.2 Former non‐users of urban green space

In all, 85 people indicated they had never used green spaces before the restrictions period (first row in Table 2), comprising 8.5% of participants. Of the 85 non‐users, 14 (16.5%) began using green spaces *once every two weeks*, 3 (3.5%) used them *once a week* and so on (see Table 2 for complete breakdown). Of these non‐users, 47 (55.3%) continued not to use green spaces, meaning that just under half of the non‐users began using green spaces for the first time during the restrictions period.

**TABLE 2:**
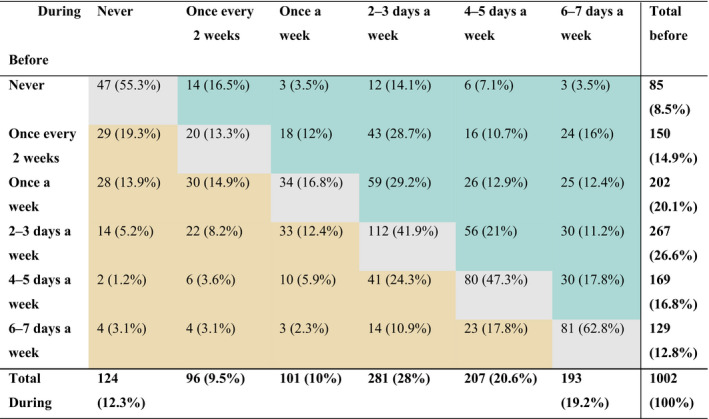
Change in use of urban green spaces before and during the restrictions period. Numbers in grey cells forming a diagonal depict participants whose use remained unchanged, green and brown cells depict participants who increased and decreased their usage, respectively, comparing usage before and during the restrictions period. Totals from rows and columns correspond to totals for each category of use before and during the restrictions period, respectively. Percentages in bold brackets represent the proportion of people from the total of participants based on their use before and during the restrictions period. Percentages in brackets represent the proportion of people based on their use before the restrictions period

### 3.3 Users who reduced or increased their use of urban green spaces

More than one‐third (36.5%) of the sample population *increased* their use of urban green spaces during the restrictions period (white cells to the right of the diagonal in Table 2). Across the different categories of frequency of use (i.e. never, once every 2 weeks, etc.), we found that between 17.8% and 67.4% of people increased the use of green spaces during the restriction period. In contrast, less than one‐third of the sample population *reduced* their use of green spaces (white cells at the left of the diagonal in Table 2). Decreases in green space use were largely a drop of only one category of use. For instance, one‐quarter (24.3%) of those who used urban green spaces 4**–**5 days a week decreased their use to 2**–**3 days a week.

### 3.4 Nature‐relatedness and change in use of urban green spaces

**FIGURE 1:**
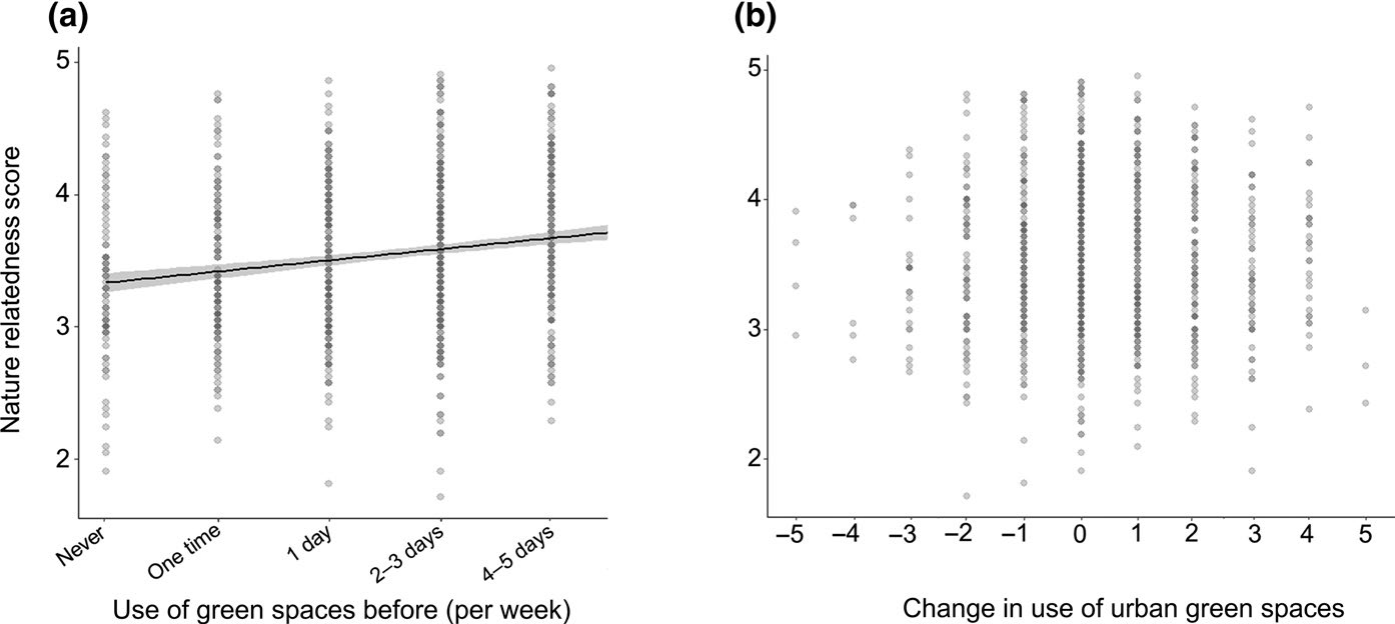
Participants' nature‐relatedness scores and (a) weekly urban green space use before the restrictions period and and (b) changes in urban green space use. Higher nature‐relatedness score indicates a stronger connection to nature (5 = high nature‐relatedness; 4,3 = moderate nature‐relatedness; 2,1 = low nature‐relatedness). Urban green space use change is represented by numbers (−5 to +5). See Table 1 caption or methods section for explanation of how the change variable is calculated

One‐third of the former non‐users had markedly low nature‐relatedness (nature‐relatedness score ≤ 3) and two‐thirds were moderate‐to‐strongly nature‐related (nature‐relatedness score ≥ 3; Figure 1a). Over half of the people who increased their green space use were moderately nature‐related (nature‐relatedness score = 3 > 4), one‐quarter was strongly nature‐related, and less than one‐quarter were weakly nature‐related (Figure 1b). Over two‐thirds of the participants that reduced their use were moderately nature‐related, only 17% were strongly nature‐related and less than one‐quarter were weakly nature‐related.

### 3.5 Predictors of change in green space use of urban green spaces

**FIGURE 2:**
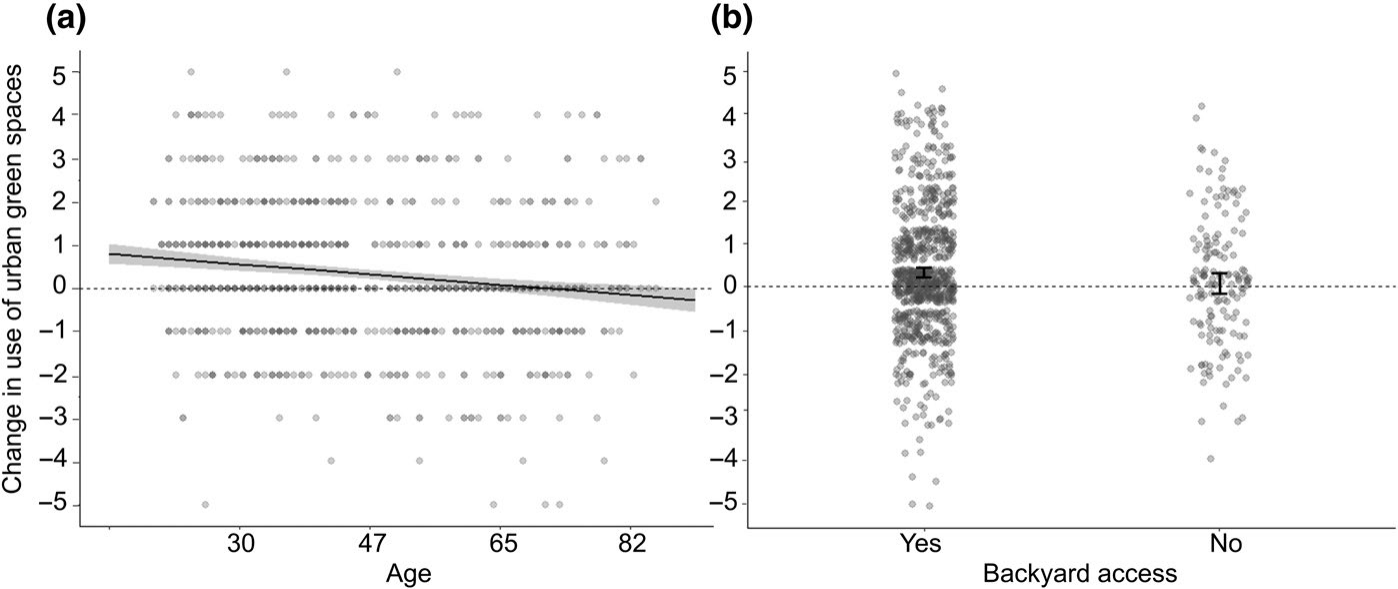
Association between (a) participant's age and change in urban green space use (black line with shading indicates the estimated regression model with 95% confidence interval); (b) backyard access and change in urban green space use (error bars indicates the 95% confidence interval estimated by the generalised linear mixed model). Urban green space use change is represented with numbers (−5 to +5). See Table 1 caption or methods section for explanation of how the change variable is calculated

We did not find a clear single model that explained green space use change from before to during the restrictions period. Within the top candidate models, the best model with the smallest AIC only included age (A) and backyard access (B) with an Akaike weight of 0.153 (Table 3). This can be interpreted that there is a 15.3% chance that A + B is the best‐approximating model describing the data given the candidate models. Notably, age is the only variable that appears in all models of the 85% confidence set, indicating its clear importance in explaining the variation in green space use change. Six models had delta AIC values below 2.0 and both age and backyard access were included in most (all for age, and five for backyard access) of these six models. The estimated coefficients indicated that age was negatively associated with changes in green space use while the accessibility to a backyard was positively associated with changes in green space use (Figure 2a,b). The results from the cumulative link mixed‐effects model were similar; both age and backyard access showed a significant association with changes in green space use (Table S1).

**TABLE 3:** 85% confidence set of best‐ranked regression models (the 17 models whose cumulative Akaike weight was ≤0.85) examining the effect of explanatory variables on changes in urban green space use in Brisbane, Australia. Gender (G), age (A), income (I), nature‐relatedness (NR), backyard access (B) and green space availability within a radius of 300 m (GS). AIC for the null model was 3,523.6

Explanatory variable	*df*	AIC	logLik	Delta AIC	Weight	Cum. weight
A + B	5	3,503.93	−1,746.93	0.00	0.15	0.15
A + NR + B	6	3,505.11	−1,746.51	1.17	0.08	0.23
A + GS + B	6	3,505.11	−1,746.51	1.18	0.08	0.32
A + I + B	6	3,505.74	−1,746.82	1.81	0.06	0.38
A + G + B	6	3,505.78	−1,746.84	1.84	0.06	0.44
A	4	3,505.83	−1,748.89	1.89	0.05	0.50
A + GS + NR + B	7	3,506.30	−1,746.09	2.36	0.04	0.55
A + G + NR + B	7	3,506.89	−1,746.38	2.95	0.03	0.58
A + I + NR + B	7	3,506.91	−1,746.39	2.97	0.03	0.62
A + GS + G + B	7	3,506.97	−1,746.42	3.03	0.03	0.65
A + GS + I + B	7	3,506.98	−1,746.43	3.04	0.03	0.68
A + GS	5	3,507.08	−1,748.51	3.14	0.03	0.72
A + NR	5	3,507.25	−1,748.59	3.31	0.02	0.75
A + I	5	3,507.52	−1,748.73	3.59	0.02	0.77
A + G + I + B	7	3,507.55	−1,746.71	3.61	0.02	0.80
A + G	5	3,507.70	−1,748.81	3.76	0.023	0.82
A + GS + G + NR + B	8	3,508.09	−1,745.96	4.15	0.019	0.84

**FIGURE 3:**
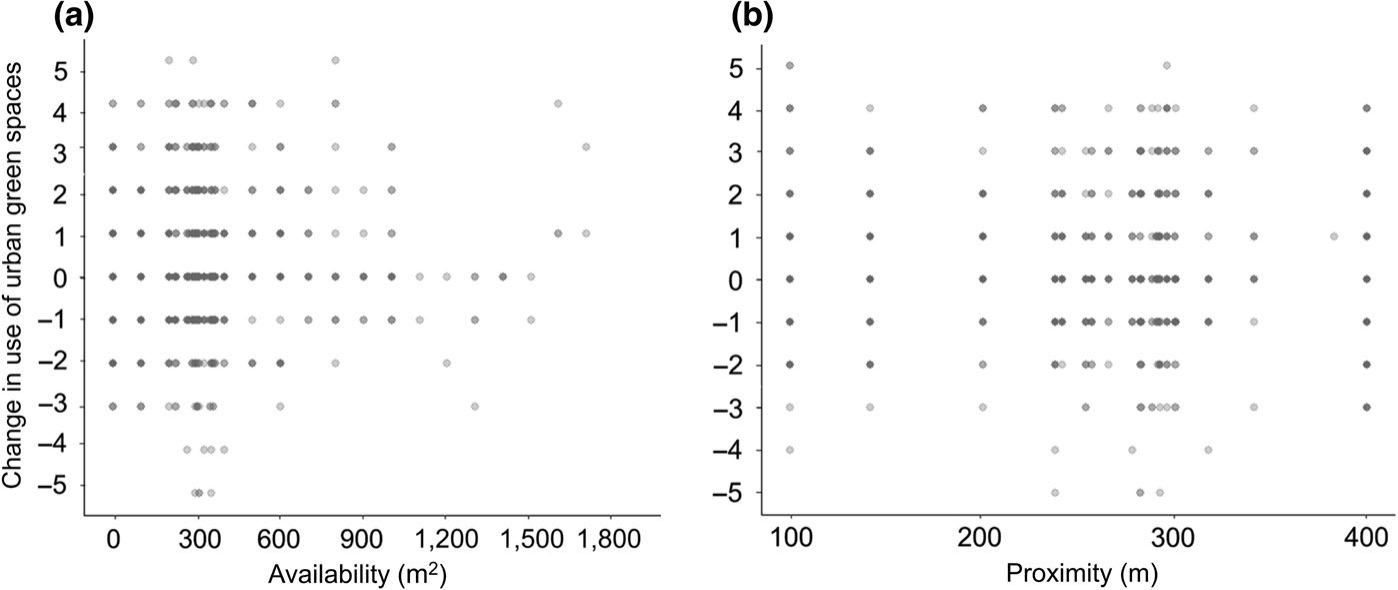
Urban green space (a) availability within 300 m radius to participant's residences and change in use and (b) proximity to participant's residences and change in use. Urban green space use change is represented with numbers (−5 to +5). See Table 1 caption and methods section for explanation of how the change variable is calculated

We found that the availability of green space did not influence change in use during the restrictions period (Figure 3a). Most participants have an average of 306 m^2^ of urban green spaces surrounding their residences within a radius of 300 m and live at an average distance of 279 m from these (Figure 3a,b). At the lower end of green space availability (200–400 m^2^), there is a similar proportion of people decreasing (15%) or increasing (19%) urban green space use. Although participants in the areas with the highest green space availability (>500 m^2^) tended to maintain or increase their frequency of use, this variable did not appear as significant in our models.

### 3.6 Reasons for using urban green during the restrictions period

**FIGURE 4:**
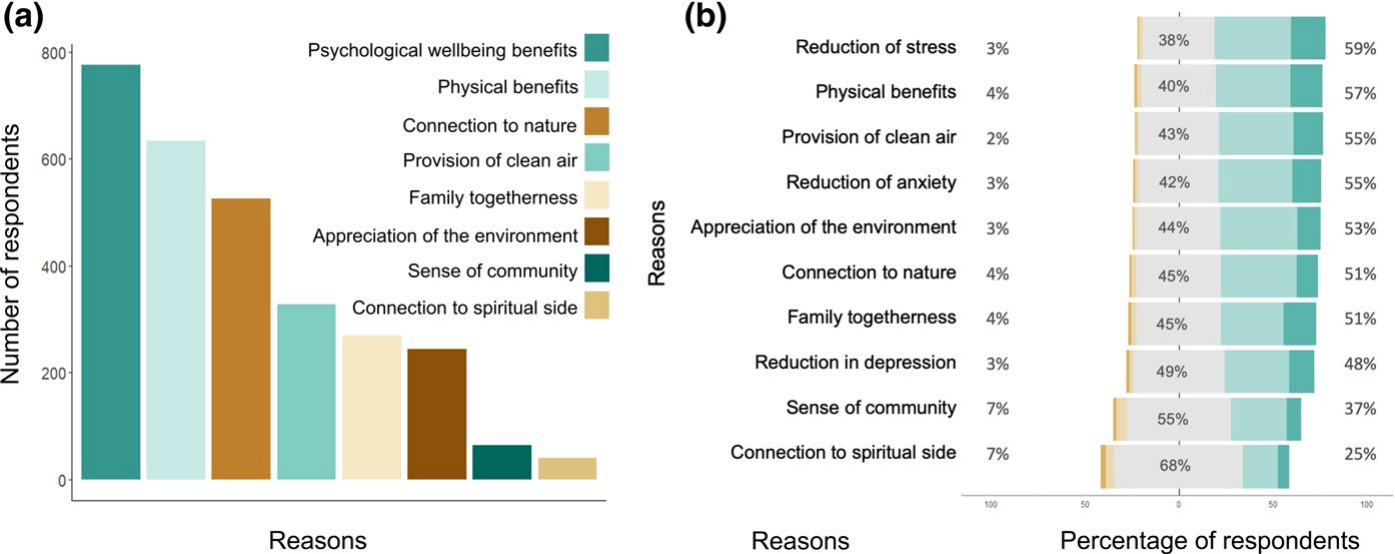
(a) Reasons for using urban green during the restrictions period, represented by differing colours and (b) Change in the reasons for using urban green during the restrictions period in comparison with before the restrictions were put in place, with numbers on the left and brown bars indicating reasons that became *less to much less important*, grey meaning *no change*, and numbers on the right and green colours indicating reasons that became *more to much more important*

People cited a broad range of the benefits obtained from visiting urban green spaces, with the top reason being *psychological well‐being benefits* (77.4%; Figure 4a) followed by *physical benefits* (62.8%), and *connection to nature* 52.4%, suggesting that visits were mostly perceived to improve general personal well‐being. Visiting green spaces for the *provision of clean air* was the main reason for 32.7% of the sample population while *sense of community* was perceived as an important reason for only 6.4% of participants.

There were marked increases in importance across almost all the benefits for visiting green spaces during the restrictions period (Figure 4b). The two exceptions to this were *sense of community* and *connection to spiritual side*, given that only 37% and 25% of participants, respectively, reported an increase in importance during the restriction period than before it (Figure 4b). Perhaps tellingly, two of the variables that form *psychological well‐being benefits*, reduction of stress and reduction of anxiety, were rated among the most important reasons for green space visitation during the restrictions period by 59% and 55% of people, respectively, and also physical benefits by 57% of participants, with these three benefits representing the biggest increase in ratings for any of the benefits of visiting green spaces (Figure 4b). In contrast to those who increased or decreased their use of green space during the restrictions period, the former non‐green space users cited the physical benefits provided by urban green spaces as their top reason for initiating visits.

## 4 DISCUSSION

Our results indicate that there were substantial changes in people's usage and perceptions of urban green spaces during the COVID‐19 pandemic restrictions period in Brisbane. These changes occurred across a broad range of socio‐demographic groups. Two‐thirds of the sample population reported changing their use of green spaces and also a significant change in the importance of their reasons for use. This suggests that people did not necessarily visited green spaces for a specific reason, but rather to accrue a higher quantity of the full range of benefits that they normally perceive from visiting green spaces. More broadly, almost 80% of participants strongly perceived that psychological well‐being could have been improved by connecting with nature and spending time in urban green spaces during a stressful moment. Echoing the findings of Marselle et al. (2019), it is possible that urban green spaces lessened some of the deleterious pandemic‐related impacts, since reduction of self‐reported stress, anxiety and depression were the main reason for visiting urban green spaces in Brisbane. Our results differed from a study conducted in Chengdu, China (Xie et al., 2020), where they found a decrease in green space weekly visit frequency, and found no significant increases in positive psychological effects after visiting urban green spaces. This could stem from differences in the severity of restrictions between the two cities. Chengdu's government strongly recommended people to use masks and to avoid enclosed and crowded places, such as public transport and gatherings (The People, 2020), whereas restrictions in Brisbane, instead of just being urged to, people were demanded to maintain social distancing and no social gatherings were allowed. Our reported increase in green space use mirrors that by Venter et al. (2020), who estimated that outdoor recreational activity in Norway increased by 291% during the restrictions period compared to a 3‐year baseline average, even after controlling for seasonality changes. Our results suggest that green spaces have a crucial role in maintaining health and well‐being during stressful periods, and particularly during the restrictions period when levels of stress, anxiety and depression were moderate to high (Salari et al., 2020).

Urban green spaces are also recognised for their role in generating social capital and reducing social isolation (Dinnie et al., 2013; Haq, 2011; Lo & Jim, 2010). However, during the restrictions period, our results show that people's reasons for use of green spaces were chiefly for the personal benefits rather than community cohesion benefits, perhaps because contact between individuals was actively discouraged to contain the disease outbreak (Australian Government Department of Health, 2020a) and also as a result of concern about spreading or contracting the disease through contact with other people (Ho et al., 2020). During the restrictions period in Brisbane, people could only use urban green spaces for recreational purposes and were limited to their immediate neighbourhood with no more than one person from the same household (Australian Government Department of Health, 2020a). Our results contrast with those of Xie et al. (2020), where survey participants chiefly visited urban green spaces to meet their social needs during the pandemic‐related mobility restrictions period although people were encouraged to minimise social interaction and avoid crowded places (The People, 2020).

Our results revealed that as long as urban green spaces are available at ‘walking distance’ (within 300 m), the amount did not influence participant's use change. This means that accessible urban green spaces, regardless of their size and amount, may have facilitated the restorative benefits provided by urban ecosystems during the restrictions period. Some studies show that a higher proportion of green space at a ‘walking distance’ translates into increased physical benefits (Giles‐Corti et al., 2005) and a community's health and well‐being (Annerstedt van den Bosch et al., 2015). Other studies have shown that pocket parks and other small green spaces may provide health benefits, mainly through psychological restoration (Kaplan et al., 1998) or recreation (Baur & Tynon, 2010). Our finding is particularly important because it indicates that, regardless of the amount of green space, people still visited them to obtain the various benefits provided, in particular psychological restoration. However, it is important to notice that in densified neighbourhoods, green spaces available at ‘walking distance’ are likely to reach their capacity and become overcrowded (Campbell, 2020) causing discomfort and health risks for certain groups.

Our results indicate that the elderly, the group who are the most at risk of contracting severe complications from COVID‐19 (Australian Government Department of Health, 2020b), were less likely to increase their green space usage, potentially as a result of fear of infection (de Leo & Trabucchi, 2020) making them more susceptible to the potential negative effects of social isolation, fear of the infection, frustration, boredom, inadequate information and financial loss (Douglas et al., 2020). Santini et al. (2020) demonstrated that social disconnection puts older adults at greater risk of depression and anxiety; thus, sufficient provision of nearby green space in areas where a high density of older adults live might help ensure that all residents' needs can be met equitably.

Due to the fear of spreading and contracting the virus discussed by Ho et al. (2020), one might have assumed that those with access to a backyard might have compensated urban green spaces visits by spending more time in their backyards during the restrictions period rather than venturing out. However, we found that people with backyard access were more likely to visit urban green spaces than those without access during the restrictions period. This result is mirrored by Lin et al. (2014) who demonstrated that green spaces users in Brisbane tended to spend more time in their backyards compared to non‐green space users. In this respect, we echo the conclusion of Lin et al. (2014), who cautioned that urban green spaces are not substitutable with backyard access. On the contrary, it indicates that the provision of urban green spaces and the benefits these provide should be strategically allocated, and perhaps focus on where they could help meet the needs of the most vulnerable during times of stress.

We initially hypothesised that people more oriented to nature would be more likely to use urban green spaces than those with a weaker connection to nature. However, our results suggest that regardless of nature‐relatedness, people changed their use of urban green spaces. Lin et al. (2014) showed that urban dwellers were motivated to use urban green spaces more by their orientation towards nature than the distance of urban green spaces to their residences, but our results suggest that during a time of elevated stress, usage of green space are possibly motivated by factors other than orientation to nature. The COVID‐19 pandemic‐related lockdowns and restrictions were simultaneously experienced by the entire Brisbane population causing health effects on people (Douglas et al., 2020), and we conclude that increased green space use, for some people, was a way to mitigate some of these impacts regardless of nature‐relatedness or former frequency of use.

A critical next step is longitudinal research to understand whether the change in frequency of use and perceptions of urban green spaces (for physical and mental well‐being) remains a sustained outcome of the COVID‐19 pandemic. It is possible that the effects of the COVID‐19 pandemic on the change in use and perception of urban green spaces may vary by context. For example, residents of low‐ and middle‐income countries may face differences in the quality and quantity of benefits derived from green spaces and accessibility to green spaces, compared to those in wealthier countries (Amano et al., 2018). As such, future research might consider these differences and conduct follow‐up surveys to estimate whether there was a change in urban green space use and perceptions during the COVID‐19 restrictions period across different contexts or countries. Another important future step is to strategically plan for the availability and proximity of urban green spaces in line with population density to avoid overcrowding (Campbell, 2020) and ensure adequate provision of ecosystems benefits. Lastly, we recommend exploring the characteristics of urban green spaces that can be optimised for stress recovery and result in use change patterns during moments of stress.

A study limitation is the retrospective nature of the data, given that participants reported their frequency of use and perceptions of the benefits of green spaces before and after the restrictions period 30–90 days after their use. This means that there may be some inaccuracy in people's reports due to the memory recall errors. Prior research has examined the effects of the length of reference periods (time frame in which participants are asked to recall activities or experiences) on memory and recall (Gryczynski et al., 2015; Winkielman et al., 1998). Ayhan and Isiksal (2004) claim that the larger the reference period, the stronger the memory error effect. Furthermore, future research can reduce the effects of recall errors by conducting a prospective study.

Our results suggest that urban green spaces are valuable urban settings that can support different forms of restoration during a global health crisis. Urban green infrastructure is not only a keystone for individual and community resilience to moments of stress in life but also provides other forms of social and environmental resilience, such as food security (Khumalo & Sibanda, 2019) and acts as a fundamental ecological structure for climate change adaptation such as flood control (Carter et al., 2018), heat stress reduction (Ranagalage et al., 2020) and air quality regulation (Lafortezza et al., 2009). Furthermore, given the positive association of urban nature with improved public health, it can also be translated into public economic benefits due to the reduction in the cost of healthcare (Natural Capital Committee, 2015); however, these benefits can be context‐dependent (Amano et al., 2018). The COVID‐19 pandemic shed light on the need to prioritise urban ecosystem services in the planning agenda and also the need to better understand the dynamics of people's uptake of these services not only under *business as usual* but also under challenging circumstances to maximise the benefits of green infrastructure. For that reason, in the post‐pandemic era, making a healthy urban environment that incorporates nature‐based solutions could make cities more resilient communities to the challenges of the twenty‐first century.

## CONFLICTS OF INTEREST

The authors declare no conflict of interest.

## AUTHORS' CONTRIBUTIONS

Conceptualisation: V.B.‐E. and R.A.F.; Formal analysis: V.B.‐E., A.F.S.C. and T.A.; Funding acquisition: R.A.F.; Methodology: V.B.‐E., A.F.S.C., T.A. and R.A.F.; Writing—original draft: V.B.‐E.; Writing—review and editing: V.B.‐E., A.F.S.C., T.A, R.R.Y.O., K.F. and R.A.F.

## Supporting information

Supplementary Material

Supplementary Material

## Data Availability

As per our agreement with the University of Queensland Ethics office and due to the confidentiality of the data, we are unable to make it publicly available.
