# Supplementary material for: Urban green space use during a time of stress: A case study during the COVID‐19 pandemic in Brisbane, Australia
Source: People Nat (Hoboken). 2021 May 26;3(3):597–609. doi: 10.1002/pan3.10218 (PMC8207087; doi:10.1002/pan3.10218)
Supplement: Supplementary file 1 — Supplementary Material [file PAN3-3-597-s001.pdf]

PEOPLE AND NATURE

Green refuges in the concrete jungle during the COVID-19 lockdown

Violeta Berdejo-Espinola, Andrés F. Suarez Castro, Tatsuya Amano, Kelly S. Fielding, Rachel Rui Ying Oh, Richard A. Fuller

Since early 2020, after the outbreak of the novel coronavirus causing the COVID-19 disease, lockdowns, social distancing rules, and facemasks became the new normal in our lives. The COVID-19 pandemic has highlighted the importance of public green spaces in cities, such as parks, nature reserves, riversides, and beaches as a refuge for urban residents, providing a range of health and other benefits.

We asked over 1000 people about how often and why they used urban green spaces before and during the lockdown, compared to before the restrictions were imposed. We found that many people changed their green space use, with over one-third of respondents increasing and less than one-third decreasing their green space use. People's reasons to visit green spaces significantly changed in importance during lockdown, with relaxation, exercise, and interacting with nature being the top three priorities. Overall, respondents' reasons for use of green spaces during lockdown were chiefly for the personal benefits rather than the opportunity to socialise with other people. We also found that younger people and those with backyards were more likely to increase their visits to green space.

Sufficient provision of nearby green space in areas where a high density of older adults live might help ensure that all residents' needs can be met and avoid jeopardising the wellbeing of the elderly in this time of crisis. Our findings emphasized the role of green spaces and the benefits these provide in enhancing urban resilience which is becoming increasingly important in our busy and changing lives.

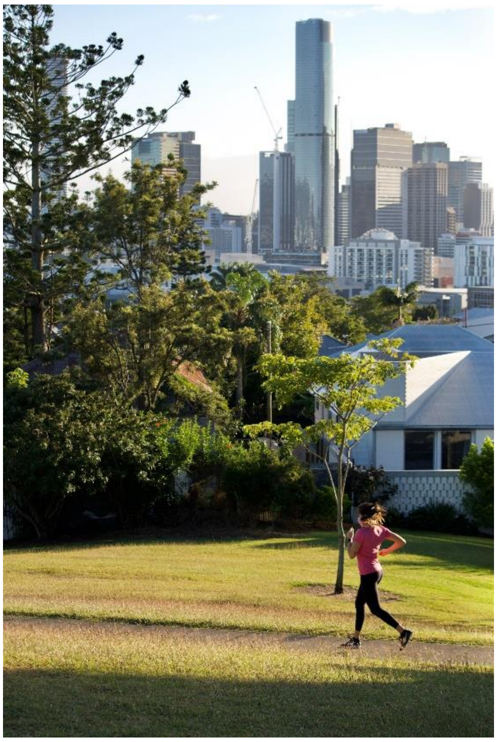

Urban green spaces as a nature-based coping mechanism during times of stress. Copyright: Christopher O'Bryan. Location: Highgate Hill Park, Brisbane, Australia.
