# Supplementary material for: Urban green space use during a time of stress: A case study during the COVID‐19 pandemic in Brisbane, Australia
Source: People Nat (Hoboken). 2021 May 26;3(3):597–609. doi: 10.1002/pan3.10218 (PMC8207087; doi:10.1002/pan3.10218)
Supplement: Supplementary file 2 — Supplementary Material [file PAN3-3-597-s002.docx]

**Supporting Information**

1. Survey delivered to Brisbane residents.

Participant Information Sheet

Monitoring use and perception of urban green spaces during the COVID-19 pandemic: a case study in Brisbane, Australia

The purpose of this study is to examine whether people’s usage and perceptions of urban green spaces have changed during the COVID-19 pandemic. The pandemic has provided a unique opportunity not only to quantify changes in the urban green spaces use under controlled conditions but also to gather information about people’s motivations for visiting green spaces. This information is key to understand, to what extent urban green spaces are a part of our arsenal of defences against deleterious impacts from the spread of the pandemic itself, and the implementation of pandemic-related restrictions.

What is involved?

Participation in this study is entirely online and will take approximately 15 minutes and the survey can be undertaken at a time and place that is convenient to you.

Participation and withdrawal

Participation in this study is completely voluntary and you are free to withdraw from this study at any time without prejudice or penalty. If you wish to withdraw, simply stop completing the survey and exit the website. Any data that is collected up to that point will not be used in the study. Feel free to ask any questions about the research (contact the project coordinator).

Risks

Participation in this study should involve no physical or mental discomfort, and no risks beyond those of everyday living. If, however, you should find any question to be invasive or offensive, you are free to omit answering or participating in that aspect of the study.

Confidentiality and security of data

Your responses to the survey are anonymous; no identifying information will be collected. You will be asked to indicate where you live (for example, provide your address or the nearest street corner) to help characterize the green spaces in your neighbourhood. Once this characterization is done, any address information will be destroyed, and it will not be passed on to anyone else. All other data will be stored on password protected computers and only members of the research team will have access to the data. Because all data is non-identifiable, it cannot be linked to individual participants and data will only be presented as summaries of overall responses. The data you provide will only be used for the specific research purposes of this study.

Benefits of your participation in the study:

The data from the survey will shed light on the role of urban green spaces to people during times of stressful life events and point to the value of the human connection with nature as a mechanism for psychological resilience to the impacts of a catastrophic event providing insights into multiple disciplines.

Consent form

Please take the time to read the project information that is provided on the previous page. Your participation is voluntary, and you can choose to withdraw at any point. You will not be asked to give your name so any information you provide is completely anonymous. Should you wish to clarify any aspect of your potential participation or need more information you can also speak directly to a lead researcher before agreeing or disagreeing to take part in the evaluation.

If you understand the purpose of the research project and the nature of your involvement, then please complete the following:

- I have read the information provided about the research project and understand the nature of my involvement. I understand any information I provide is completely confidential. I agree to take part and understand I can withdraw at any time without prejudice or penalty.
- I am over 18 years of age.

Survey

| **1. Socio-demographic information** | ***COVID-19 restrictions period:*** *from 23^rd^ of March, when lockdown restrictions were first put in place until 2^nd^ May 2020, when the Queensland Government began easing restrictions on movement (e.g.: people were allowed to travel for recreation within 50km).* | |
| --- | --- | --- |
|  | 1. What is your year of birth? | Year: |
|  | 2. What is your gender? | Male/Female/Other |
|  | 3. What suburb do you live in? | BRIS LGA SUBURB LIST + other |
|  | 4. Before tax, what is the total of all wages/salaries, government benefits, pensions, allowances and other income you usually received *before the COVID-19 restrictions period*? (please tick one). | $2000 or more a week ($104,000 or more per year)  $1,500 - $1,999 a week ($78,000 -$103,999 per year  $1,250 - $1,499 a week ($65,000 – $77,999 per year)  $1,000 - $1,249 a week ($52,000 - $64,999 per year)  $800 - $999 a week ($41,600 - $51,999 per year)  $600 - $799 a week ($32,200 - $41,599 per year)  $400 - $599 a week ($20,800 - $31,199 per year)  $300 - $399 a week ($15,600 - $20,799 per year)  $200 - $299 a week ($10,400 - $15,599 per year)  $1 - $199 a week ($1 - $10,399 per year)  Nil or negative income |
|  | 5. Do you have access to a private garden or a backyard? | Yes/No |
|  | 6. What is the level of the highest qualification or schooling year you have completed? (please tick one). | Year 12 or equivalent  Year 11 or equivalent  Year 10 or equivalent  Year 9 or equivalent  Year 8 or below  Certificate 1, 2, 3, 4  Trade certificate  Bachelor degree  Associate diploma  Advanced diploma  Post-graduate degree  Other (please specify)___ |
|  | 7. Do you speak a language other than English at home? | Yes/No  If yes, what other language do you speak most often? |
|  | 8. What is your address to the nearest ten houses, e.g. 90- 100 Manly Road or the nearest junction of two streets to your place of residence? | Address or  Street 1/Street 2  Suburb  Postcode  Prefer not to say |
|  | 9. Did you have to move residences due to the circumstances of the COVID-19 pandemic?  9a. Please provide the address to the nearest ten houses, e.g. 90- 100 Manly Road or the nearest junction of two streets to your place of previous residence. |  |
|  | 10. Including you, how many people live at your home who are: (please write in number) | Under 16 years __________________ 16 years and over ________________ |
|  | 11. How many school aged children were being kept at home during the COVID-19 restrictions period? | Number of children:  NA |
|  | 12. Did you work from home during the *COVID-19 restrictions period* because of the lockdown restrictions? | Yes/No/Other |
|  | 13. How many days a week do you normally do paid work? | 0,1, 2, 3, 4, 5,6 ,7 |
|  | 14. In a normal week, about how many hours do you do paid work? | No time  5 hours or less  6-10 hours  11-20 hours  21-30 hours  31-40 hours  41-50 hours  51-60 hours  61-70 hours  71+ hours |

| **2. Outdoor green spaces use and perceptions** | ***This next section is about outdoor green spaces. Outdoor green spaces*** *include parks, bushlands, picnic areas, riversides, beaches.* | |
| --- | --- | --- |
|  | ***Please think about the COVID-19 restrictions period:*** *from 23^rd^ of March, when lockdown restrictions were first put in place until 2^nd^ May 2020, when the Queensland Government began easing restrictions on movement (e.g.: people were allowed to travel for recreation within 50km).* | |
|  | **During COVID-19 restrictions period** | |
|  | 15. If you have a backyard, how often did you spend time in it **during** the COVID-19 restrictions period? | Never  Once a week  2-3 days a week  4-5 days a week  6-7 days a week |
|  | 16. About how often **during** the COVID-19 restrictions period did you visit or pass through an outdoor green space for any reason? | Never  Once every two weeks  Once a week  2-3 days a week  4-5 days a week  6-7 days a week |
|  | 17. **During** the COVID-19 restrictions period, what outdoor green spaces did you visit or pass through?  (Please list up to seven of the places you visited for the longest period of time).  Please provide either the name, location, or some identifiable landmark.  17a. Can you estimate the total time you spent there? | 1-29 minutes  30 minutes to -1 hour  >1-2 hours  >2-3 hours  >3-4 hours  >4 hours |
|  | 18. Please tick the main activities that you undertook while in outdoor green spaces **during** the COVID-19 restrictions period. | Relax and unwind  Exercise/Sports  Being in nature  Walking the dog  Pass through when walking/cycling to work/shop/others  Other (please specify) |
|  | 19. Please list the three most important reasons why you visit an outdoor green space normally. | Physical health benefits (weight, cardiovascular)  Reduction of stress  Reduction anxiety  Reduction in depression  Connection to nature  Connection to spiritual side  Appreciation of the environment  Family togetherness  Provision of clean air  Sense of community |
|  | 20. Have any of the reasons listed below become more or less important **during** the COVID-19 restrictions period? On a scale:  - Much less important  - Less important  - No change  - More important  - Much more important. | Physical health benefits (weight, cardiovascular)  Reduction of stress  Reduction of anxiety  Reduction in depression  Connection to nature  Connection to spiritual side  Appreciation of the environment  Family togetherness  Provision of clean air  Sense of community |

|  | **Before COVID-19 restrictions period** | |
| --- | --- | --- |
|  | Now we are going to ask some questions about how you used green spaces before COVID-19 restrictions. Please think about before COVID-19 for these questions. | |
|  | 21. If you have a backyard, how often did you spend time in it **before** the COVID-19 restrictions period? | Never  Once a week  2-3 days a week  4-5 days a week  6-7 days a week |
|  | 22. About how often **before** the COVID-19 restrictions period did you visit or pass-through outdoor green spaces for any reason? | Never  Once every two weeks  Once a week  2-3 days a week  4-5 days a week  6-7 days a week |
|  | 23. **Before** the COVID-19 restrictions period, what outdoor green spaces did you visit or pass through? (Please list up to seven of the places you visited for the longest period of time).  Please provide either the name, location, or some identifiable landmark.  23a. Can you estimate the total time you spent there per visit in a typical visit/day? | 1-29 minutes  30 minutes to -1 hour  >1-2 hours  >2-3 hours  >3-4 hours  >4 hours |
|  | 24. Please tick the main activities that you undertook while in an outdoor green space **before** the COVID-19 restrictions period. | Relax and unwind  Exercise/Sports  Being in nature  Walking the dog  Pass through when walking/cycling to work/shop/others  Other (please specify) |

|  | **In general** | |
| --- | --- | --- |
|  | 25. What would be your preferred distance to travel to an outdoor green space on a typical day? | Within 3 blocks  3 to 4 blocks  4 to 6 blocks  6 to 8 blocks  More than 8 blocks |
|  | 26. How often do any of the following factors prevent you from spending time in an outdoor green space?  - Never  - Sometimes  - Often  - Most of the time. | I prefer to do other indoor activities  A lack of time  Magpies or other birds  Snakes  Biting insects  Too hot/cold  Worried about getting too much sun  Not safe during the day  Not safe at night  Not appropriate facilities  Facilities are too far away or inaccessible  A lack of transport  Not safe during my journey to the green space  Poor health prevents me from getting outdoors  Green space is not well maintained |
|  | 27. In the last week, how many times did you do any moderate or vigorous physical activity that made you breathe harder or puff or pant?  - Not at all  - 1 time  - 2-3 times  - 4-6 times  - 5-6 times  - 7 or more times | Individual pursuit, e.g., jogging, cycling, swimming, tennis, surfing etc.  Walking for more than 30 minutes at a time  Other activities, e.g., gardening, heavy work around the home or at work etc. |

| **3. Nature relatedness** | 28. Please reflect on the period **during COVID-19 restrictions. Thinking about this period**, please rate the extent to which you agree with each statement. Please tick the box that indicates how you really feel, rather than how you think “most people” feel.  On a scale:  - Disagree strongly  - Disagree a little  - Neither Agree nor Disagree  - Agree a little  - Agree strongly | I enjoy being outdoors, even in unpleasant weather.  Some species are just meant to die out or become extinct.  Humans have the right to use natural resources any way we want.  My ideal vacation spot would be a remote wilderness area.  I always think about how my actions affect the environment.  I enjoy digging in the earth and getting dirt on my hands.  My connection to nature and the environment is a part of my spirituality.  I am very aware of environmental issues.  I take notice of wildlife wherever I am.  I don’t often go out in nature.  Nothing I do will change problems in other places on the planet.  I am not separate from nature, but a part of nature.  The thought of being deep in the bush, away from civilisation, is frightening.  My feelings about nature do not affect how I live my life.  Animals, birds and plants should have fewer rights than humans.  Even in the middle of the city, I notice nature around me.  My relationship to nature is an important part of who I am.  Conservation is unnecessary because nature is strong enough to recover from any human impact.  The state of non-human species is an indicator of the future for humans.  I think a lot about the suffering of animals.  I feel very connected to all living things and the earth. |
| --- | --- | --- |

1. Table 1. Results from cumulative link mixed model examining the association between change in green space use and six explanatory variables in Brisbane, Australia. Random effects variance = 5.453645e-09, standard error = 7.38488e-05.

| **Explanatory variables** | **Estimate** | **Standard error** | | **z-value** |
| --- | --- | --- | --- | --- |
| Gender | 0.025 | 0.117 |  | 0.217 |
| Age | -0.292 | 0.059 |  | -4.937 |
| Income | 0.031 | 0.059 |  | 0.529 |
| Green space availability within  a radius of 300m | 0.024 | 0.056 |  | 0.433 |
| Nature relatedness | -0.053 | 0.058 |  | -0.922 |
| Backyard access | -0.307 | 0.161 |  | -1.897 |
